# Dual Role of HNF4α in Colorectal Adenocarcinoma During Carcinogenesis and Metastasis

**DOI:** 10.3390/cells14080599

**Published:** 2025-04-15

**Authors:** Ju Seok Kim, Kyung-Hee Kim, Jun Young Heo, Min Kyung Choi, Min-Kyung Yeo

**Affiliations:** 1 Department of Internal Medicine, Chungnam National University School of Medicine, Daejeon 34134, Republic of Korea; showsik@cnuh.co.kr; 2 Department of Pathology, Translational Immunology Institute, Chungnam National University School of Medicine, Daejeon 34134, Republic of Korea; phone330@cnu.ac.kr (K.-H.K.); mk6214@cnu.ac.kr (M.K.C.); 3 Department of Biochemistry, Chungnam National University School of Medicine, Daejeon 34134, Republic of Korea; jyheo@cnu.ac.kr; 4 System Network Inflammation Control Research Center, Chungnam National University, Daejeon 34134, Republic of Korea; 5 Department of Medical Science, Chungnam National University School of Medicine, Daejeon 34134, Republic of Korea

**Keywords:** hepatocyte nuclear factor 4α, colorectal adenocarcinoma, carcinogenesis, metastasis, Yes-associated protein

## Abstract

Hepatocyte nuclear factor 4α (HNF4α), a highly conserved member of the nuclear receptor superfamily of transcription factors, has been identified as a promising therapeutic candidate for colorectal adenocarcinoma (CRAC). This study was to investigate the significance of HNF4α in CRAC and mechanisms governing its function. The expression patterns and clinical relevance of HNF4α were evaluated in relation to nuclear factor kappa B (NF-κb), Yes-associated protein (YAP), and epithelial–mesenchymal transition markers. HNF4α exhibited upregulation during carcinogenesis compared to normal and precancerous lesions. The overexpression and inhibition of HNF4α were correlated with the modulation of CRAC cell migration and invasion, either promoting or suppressing these processes. Notably, levels of HNF4α were significantly diminished in metastatic and poorly differentiated CRAC relative to primary CRAC samples. Moreover, reduced HNF4α levels were associated with unfavorable prognostic factors. The inhibition of HNF4A induced a decrease in NF-κb protein levels, concomitant with an increase in YAP. Our results indicate a dual role of HNF4α in tumor progression, either as a promotor or inhibitor, depending on the pathologic condition of CRAC and the related signaling pathways. HNF4α exhibits a complex role, whereby its overexpression is linked to early carcinogenesis and reduced expression is associated with the progression and metastasis of CRAC.

## 1. Introduction

Hepatocyte nuclear factor 4α (HNF4α) belongs to a highly conserved nuclear receptor superfamily of transcription factors. HNF4α is designated based on its critical role in regulating hepatic morphogenesis and metabolism [1,2]. Additionally, HNF4α contributes to maintenance of the epithelial barrier, immune cell function, ion transport, and management of oxidative stress [3]. In the context of cancer, HNF4α is implicated in essential processes, such as differentiation, proliferation, invasion, migration, and apoptosis [4]. Aberrant and altered changes in HNF4α have been associated with the development of several cancer types, including hepatocellular carcinoma (HCC), renal cell carcinoma (RCC), pancreatic adenocarcinoma (PAAD), stomach adenocarcinoma (STAD), cholangiocarcinoma, and colorectal adenocarcinoma (CRAC) [5,6,7].

CRAC is the third leading cause of cancer-related deaths in men and the second highest contributor to cancer-associated mortality in the United States [8]. Surgical intervention is the primary treatment of choice, with a 5-year survival rate of approximately 80% for patients achieving a curative outcome post-surgery. However, 10–30% patients present with distant metastases at the time of surgery [9,10]. Additionally, metachronous metastases after surgical resection occur in 5–6% cases [11], highlighting the importance of therapeutic target validation for CRAC to achieve effective control of metastases. HNF4α has emerged as a potential candidate for CRAC therapy, in view of its elevated expression levels across various types of cancer. Data from The Cancer Genome Atlas Program (TCGA) and Human Protein Atlas (HPA) consistently demonstrate the upregulation of HNF4α [12]. Furthermore, as a nuclear receptor, HNF4α possesses hydrophobic ligand-binding pockets that present a valuable natural target for novel drug development [13,14,15].

Conflicting results have highlighted both the oncogenic and tumor suppressor properties of HNF4α in different cancer types. HNF4α performs a tumor suppressor function in prostate cancer [16] and in PAAD [17]. Also, HNF4α downregulation is reported to enhance drug resistance in STAD through the regulation of cell apoptosis [18]. The decreased expression of HNF4α is correlated with aggressive behavior related to the promotion of epithelial–mesenchymal transition (EMT) via the Wnt/β-catenin signaling pathway in HCC [19,20]. Conversely, HNF4α exhibits oncogenic behavior in HCC and plays a role in cancer development and metastatic tumor formation [21,22,23]. In CRAC, HNF4α is implicated in carcinogenesis and metastasis [24]. Its upregulation is associated with enhanced tumorigenesis and positively correlated with the Wnt/β-catenin pathway [25,26]. Additionally, the intestine-specific knockout of HNF4α has been shown to confer protection against inflammatory bowel disease through maintaining the intestinal mucosal barrier [27]. The biological functions and associated regulatory pathways of HNF4α have not been well characterized. This study aimed to elucidate the precise role of HNF4α and its clinical relevance in CRAC, with a focus on the underlying molecular mechanisms.

## 2. Materials and Methods

### 2.1. Cell Lines and Culture

Human colon adenocarcinoma cell lines LoVo (KCLB No. 10229), SW480 (KCLB No. 10228), COLO205 (KCLB No. 10222), HCT116 (KCLB No. 10247), HT29 (KCLB No. 30038), and Caco2 (KCLB No. 30037.1) were purchased from the Korean Cell Line Bank (KCLB, Seoul, Republic of Korea). CRAC cells were cultured in RPMI1640 medium (Hyclone, Logan, UT, USA) supplemented with 10% fetal bovine serum, 1× penicillin, and streptomycin. Cells were grown in a humidified incubator at 37 °C under an atmosphere of 5% CO_2_.

### 2.2. HNF4α Overexpression

The pCMV6-Entry vector (PS100001) and human HNF4α cDNA clone (RC217863) were purchased from Origene Technologies, Inc. (Rockville, MD, USA). Colon cancer cells were cultured in a 60 mm plate until 60–70% confluency and transfected for 6 h with either empty control pCMV6 vector or pCMV6- HNF4α using Lipofectamine 2000 (Invitrogen, Carlsbad, CA, USA), in keeping with the manufacturer’s protocol. The transfection medium was subsequently replaced with culture medium containing fetal bovine serum (FBS) and cells were harvested at 24 or 48 h after transfection.

### 2.3. HNFA siRNA Transfection

Short interference RNAs (siRNAs) targeting the HNF4α gene and negative control were synthesized by Bioneer (Daejeon, Republic of Korea). Cells were seeded in a 6-well plate until attainment of 70–80% confluence, and, the following day, transfection was performed using Lipofectamine RNAiMAX reagent (Invitrogen) with negative control siRNA and HNF4α siRNA, in accordance with the manufacturer’s instructions. The sequence of HNF4α siRNA was 5′-GGCCAAGUACAUCCCAGCUUU-3′ and that of control siRNA (siCtrl) was 5′-AAAGCUGGGAUGUACUUGGCC-3′.

### 2.4. Transwell Migration and Invasion and Wound-Healing Assays

Migration and invasion assays were conducted using Transwell chambers (8 µm pore size cell culture insert, Falcon, BD biosciences (BD Biosciences, San Jose, CA, USA) in 24-well plates. For the migration assay, the lower chamber was coated with 0.1% gelatin (Sigma-Aldrich, St. Louis, MO, USA). For the invasion assay, the upper chamber was coated with 2 mg/mL Matrigel (354234, Corning, NY, USA). Cells were seeded in serum-free medium in the upper chamber, while the lower chamber was supplemented with 700 µL medium containing 20% fetal bovine serum. After 24 h of incubation at 37 °C, non-migratory cells that remained in the upper chamber were removed using a cotton swab. The remaining cells were fixed in 10% formalin for 1 h and stained with 0.1% crystal violet for 30 min. Cell migration and invasion were quantified using ImageJ software (Version 1.54p) from images captured at 200× magnification in three randomly selected fields under an Olympus IX71 microscope (Olympus, Tokyo, Japan), and the average values were determined. For the wound-healing assay, cells were seeded in the plates. After starving overnight in medium supplemented with 1% FBS, the confluent monolayers were created by a linear wound by scraping. Plates were washed with PBS and cultured with a medium for 24 h. Images of the wounds were captured and the horizontal distance between the edges of the wound was measured. All experiments were performed in triplicate.

### 2.5. Western Blot Analysis

Cells were lysed in RIPA buffer (LPS, Daejeon, Republic of Korea) containing a protease inhibitor cocktail (Sigma) and a phosphatase inhibitor cocktail (Roche, Basel, Switzerland). Lysates were centrifuged at 14,000 rpm (18,472× *g*) for 20 min at 4 °C to extract proteins. Equal amounts of total protein from cell lysates were separated via 10% SDS-PAGE and transferred to polyvinylidene difluoride membranes (Millipore, Burlington, MA, USA). Membranes were incubated in blocking solution with gentle agitation for 1 h, followed by overnight incubation at 4 °C with the appropriate primary antibody. After washing three times for 10 min each with TBST, the membrane was incubated at room temperature with horseradish peroxidase-conjugated secondary antibodies, specifically, anti-rabbit IgG (1:5000, 7074, Cell Signaling Technology, Danvers, MA, USA) and anti-mouse IgG (1:5000, 7076, Cell Signaling Technology). The membrane was re-washed three times for 10 min each with TBST. Protein expression was detected using the Advansta Western Bright Sirius Western blot kit (Advansta, CA, USA). The following antibodies were employed: HNF4α (1:1000, MA5-14891, Invitrogen), GAPDH (1:5000, sc-47724, Santa Cruz Biotechnology, Dallas, TX, USA), phosphor-NF-κb p65 (p-NF-κb p65) (3033, Cell Signaling), NF-κb p65 (8242, Cell Signaling), NF-κb p105/50 (ab7549, Abcam, Cambridge, UK), NF-κb p100/52 (4882, Cell Signaling), RelB (10544, Cell Signaling), Yes-associated protein (YAP) (4912, Cell Signaling), E-cadherin (3195, Cell Signaling), EpCAM (sc-25308, Santa Cruz), N-cadherin (13116S, Cell Signaling), Vimentin (5741S, Cell signaling), and Twist (sc-81417, Santa Cruz). Tissues obtained from patients were sliced into 4 µm sections using a blade and transferred into an Eppendorf tube containing sterilized beads, followed by the addition of 700 µL ice-cold lysis buffer to the mixture. The sample was homogenized for 15–20 min using Qiagen Tissue Lyser 2 (Qiagen, Hilden, Germany). The tube was centrifuged at 14,000× *g* for 20 min at 4 °C, after which the supernatant was transferred to a new tube. The Western blots were quantified by Image J program (https://imagej.net/ij/index.html (accessed on 27 December 2024)). The relative quantification values of each tissue sample and cell line were presented as the ratio of their band value to that of GAPDH band value (Supplementary Data).

### 2.6. Immunohistochemical Staining

Paraffin-embedded tissue sections were cut at a thickness of 3 µm and deparaffinized using xylene and a graded alcohol sequence, followed by rehydration. Antigen retrieval was performed by heating the samples in a pressure cooker for 5 min using either citrate buffer, pH 6.0 (Dako, Glostrup, Denmark), or Target buffer, pH 9.0 (Dako). To block endogenous peroxidase activity, sections were treated with 3% hydrogen peroxide and pre-incubated with Protein Block serum (Dako). Tissues were incubated with primary antibodies specific for HNF4α (1:200, MA5-14891, Invitrogen), pNF-κb p65 (1:100, 44-711G, Invitrogen), and YAP (1:200, 4912S, Cell Signaling) at room temperature for 30 min. After washing, sections were incubated with the appropriate secondary antibodies (Envision+ System-HRP Labeled Polymer Anti-Rabbit, Cat. no. K4003, Dako, Agilent Technologies, Inc. Santa Clara, CA, USA, and Envision+ System-HRP Labeled Polymer Anti-Mouse, K4001, Dako, Agilent Technologies, Inc.) at room temperature for 30 min, followed by a further wash. Slides were stained with 3,3′-diaminobenzidine (DAB, Cat. no. K3468, Dako, Agilent Technologies, Inc.) and counterstained with Meyer’s hematoxylin at room temperature, followed by dehydration and mounting.

Immunohistochemical staining was assessed using light microscopy, including the intensity of staining and proportion of stained tumor cells on each slide. The intensity and proportion of staining were quantified via histo-score (H-score), categorized as four IHC levels: negative (0), weak (1+), moderate (2+), and strong (3+). In each case, the H-score (potential range of 0–300) was calculated as follows: H-score = ((1 × % weakly stained cells) + (2 × % moderately stained cells) + (3 × % strongly stained cells)) [28]. Individual samples were examined separately and scored by two pathologists (M.-K.Y. and K.-H.K.). Any discrepancies in the scores were discussed to obtain a consensus.

### 2.7. CRAC Patients and Specimens

In total, 148 patients histologically diagnosed with CRAC and subjected to curative surgical treatment were included for study. Information on patients and survival outcomes were collected from a single institute medical record. None of the patients received pre-operative chemotherapy or radiotherapy. Cancer stages were determined according to the American Joint Committee on Cancer (AJCC) staging system, 8th edition [29]. A comprehensive clinicopathological review of all cases was performed by two pathologists (M.-K.Y. and K.-H.K.). All paraffin-embedded tissue samples of CRAC patients were obtained from Chungnam National University Hospital (Daejeon, Republic of Korea) from 2002 to 2015. The most representative and viable tumor and non-tumor areas were selected and marked on hematoxylin and eosin (H&E)-stained slides. To create a tissue microarray, 3.0 mm tissue columns were obtained from the original blocks and inserted into recipient tissue array paraffin blocks (each designed to accommodate 30 holes in a column). Seven malignant polyps containing the sequence of non-neoplastic colorectal tissue–adenoma–CRAC and 19 tubular adenomas with low-grade dysplasia (TA) along with 25 matched metastatic colorectal cancer samples were evaluated. Synchronous metastasis was defined as “metastasis at the same time as the diagnosis of the primary tumor” and metachronous as “metastasis developing more than six months after the surgical removal of the first CRAC”. All specimens were provided by the national biobank of Korea, Chungnam National University Hospital (CNUH), a member of the Korea Biobank network. A flow chart of the experimental steps of our study is presented (Supplementary Figure S1).

### 2.8. Statistical Analysis

Associations of immunohistochemical and Western blot-based HNF4α expression with clinicopathologic variables were examined via Spearman rank correlation coefficient, Mann–Whitney U, and Kruskal–Wallis tests. The univariate overall and disease-free survival curves were determined by the Kaplan–Meier method using log-rank test. The multivariate survival analysis was conducted using Cox’s proportional hazard regression model. Statistical significance was defined as *p* < 0.05 (SPSS 29; SPSS Inc., Chicago, IL, USA).

## 3. Results

### 3.1. Expression Patterns of HNF4α in CRAC

**Figure 1:**
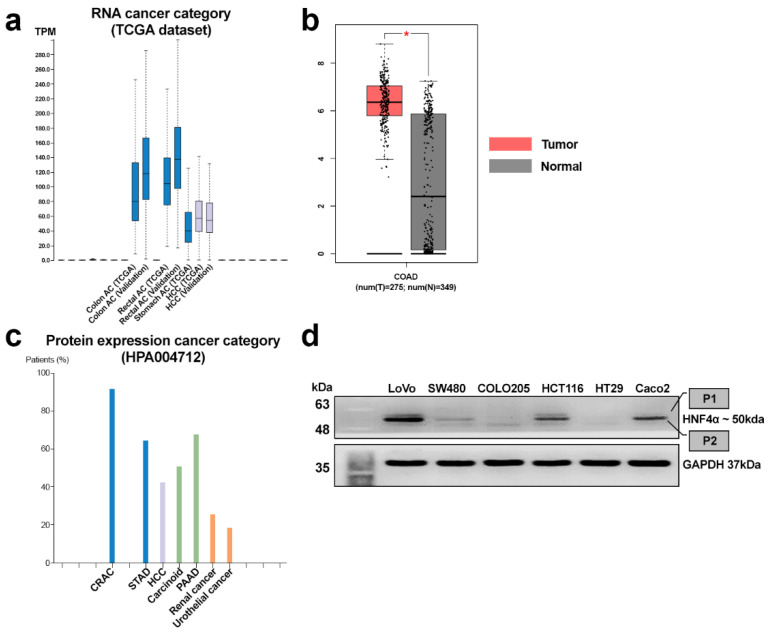
(**a**) Differential expression of HNF4α from RNA sequencing data of TCGA samples. Higher HNF4α expression was detected in colon and rectal AC (adenocarcinoma) compared to HCC. (**b**) HNF4α expression in CRAC tissues and their matched normal counterparts (* *p* < 0.01). (**c**) Protein expression of HNF4α among different cancer types from Human Protein Atlas data samples. Protein expression of HNF4α was the highest in CRAC compared to STAD and PAAD. (**d**) Immunoblot analyses of HNF4α (P1/P2) in colon cancer cell lines. Strong HNF4α expression in the LoVo, HCT116, and Caco2 cell lines, while weak or minimal HNF4α expression in the SW480, COLO205, and HT29 cell lines observed in the Western blot.

HNF4α RNA expression patterns across various cancer types derived from the public TCGA dataset (https://www.proteinatlas.org/ENSG00000101076-HNF4A/cancer (accessed on 27 December 2024)) are presented as box plots (Figure 1a). The highest HNF4α expression was observed in colon adenocarcinoma (Colon AC) and rectal adenocarcinoma (Rectal AC) relative to the other types of solid cancers, including HCC. The average fragments per kilobase of transcript per million (FPKM) of CRAC was the highest in the TCGA dataset, recorded as 43.5 (Figure 1a). HNF4α was upregulated in CRAC tumor samples compared to their matched normal counterparts (http://gepia.cancer-pku.cn/detail.php?gene=HNF4A (accessed on 27 December 2024)) (Figure 1b). When HNF4α protein expression was compared among various cancers using the HPA004712 antibody, the highest expression of HNF4α was observed in CRAC tissue samples relative to the other types of solid cancers, including STAD and PAAD. (https://www.proteinatlas.org/ENSG00000101076-HNF4A/cancer (accessed on 27 December 2024)) (Figure 1c). The majority of CRACT samples (91%, 10/11) expressed HNF4α immunohistochemical staining. Additionally, HNF4α protein levels were evaluated in six commercial colorectal cancer cell lines. HNF4α was strongly detected in LoVo, HCT116, and Caco2 cell lines, while SW480, COLO205, and HT29 cell lines showed weak or minimal HNF4α expression. HNF4α was observed as two distinct bands at ~47 kDa and ~50 kDa corresponding to P1- and P2-HNF4α, as demonstrated in previous studies (Figure 1d). LoVo, HCT116, and Caco2 cell lines displayed strong P2- and faint P1-HNF4α expression.

### 3.2. Overexpression and Knockdown of HNF4α in CRAC Cell Lines

**Figure 2:**
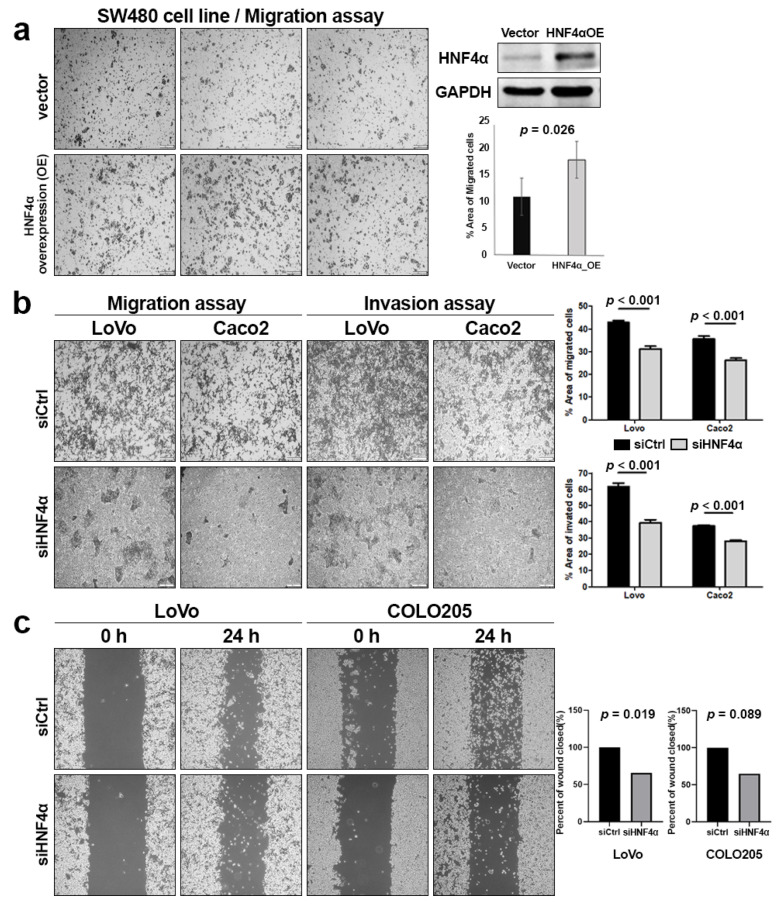
(**a**) SW480 cells expressing HNF4α displayed increased migration compared to their control counterparts. (**b**) Assessment of the migration and invasion abilities of LoVo and Caco2 cells with HNF4α inhibition using the Transwell assay. (**c**) Wound-healing assay of LoVo and COLO205 cells, indicating a decrease in the percentage of wound closure and migration rate in HFN4α-inhibited LoVo cells.

We further attempted to evaluate the effects of HNF4α regulation on colonic cancer cell lines. To validate the involvement of HNF4α in CRAC cell growth and migration, we generated an SW480 cell line with stable HNF4α overexpression. The Transwell migration assay was conducted to evaluate the potential regulatory effect of HNF4α on migration and invasion. Our results showed that HNF4α overexpression enhanced the cell migration by 1.4, 1.8, and 1.6 folds, respectively, compared with SW408 cells transfected with the control vector (*p* = 0.026) (Figure 2a).

LoVo and Caco2 cell lines were infected with lentivirus containing HNF4α-targeted shRNA, and the knockdown effect was confirmed via Western blot analysis. Transwell migration and invasion assays were employed to validate the impact of HNF4α regulation on cancer cell behavior (Figure 2b). The inhibition of HNF4α markedly suppressed the migration of LoVo and Caco2 cells by 27.3% (42.94 ± 0.49 vs. 31.23 ± 2.84, *p* < 0.000) and 26.3% (35.58 ± 1.03 vs. 26.22 ± 0.64, *p* < 0.000), respectively. The invasive rates of LoVo and Caco2 cells decreased by 36.1% (61.68 ± 1.19 vs. 39.40 ± 1.38, *p* < 0.000) and 25.1% (37.41 ± 0.66 vs. 28.02 ± 0.68, *p* < 0.000), respectively. The wound-healing assay indicated a notable decrease in wound closure and migration in the HNF4α inhibition group relative to the control group in LoVo cell lines (Figure 2c). The inhibition of HNF4α led to a decrease in migration in LoVo and COLO205 cells by 14.23% (29.38 ± 1.35 vs. 33.56 ± 0.55, *p* = 0.019) and 28.76% (24.79 ± 3.14 vs.31.92 ± 4.89, *p* = 0.089), respectively. The LoVo cells were significantly inhibited by the HNF4α inhibition. However, the COLO205 cells, which showed weak HNF4α expression, did not experience significantly suppressed migration by the HNF4α inhibition.

### 3.3. HNF4α Protein Expression Is Associated with CRAC Progression

**Figure 3:**
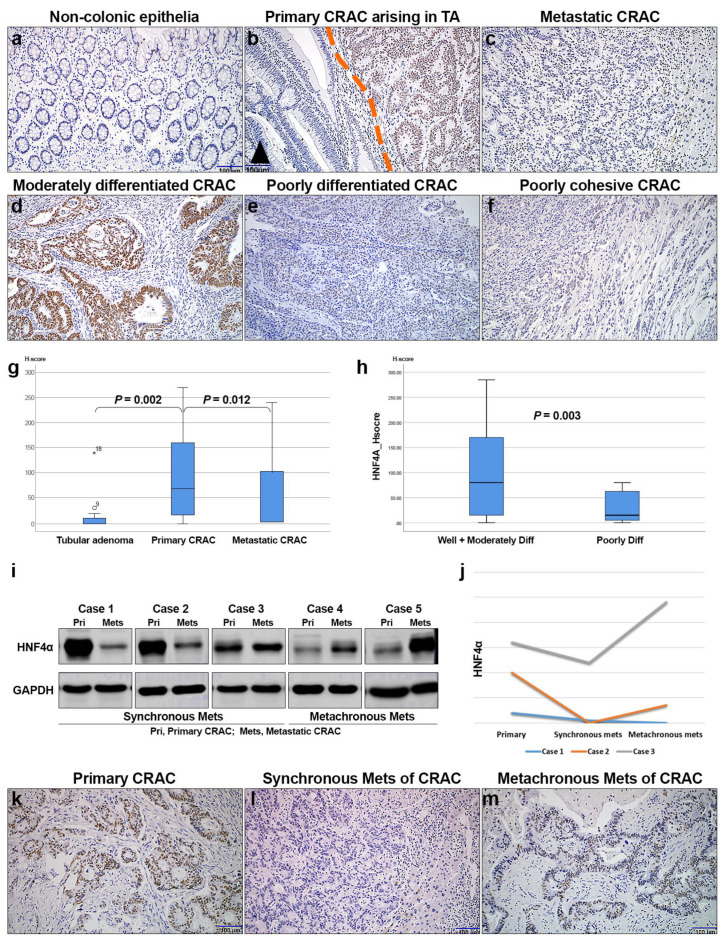
Immunohistochemical localization of HNF4α in human tissue samples of (**a**) normal colonic epithelia, (**b**) tubular adenoma (**left**) and primary CRAC (**right**), (**c**) metastatic CRAC in the liver, (**d**) moderately differentiated CRAC, (**e**) poorly differentiated CRAC, and (**f**) poorly cohesive CRAC. (**g**) Primary CRAC samples displayed higher HNF4α expression than normal epithelia and metastatic CRAC (*p* = 0.002 and *p* = 0.012). (**h**) Well- and moderately differentiated CRAC samples displayed elevated expression of HNF4α. (**i**) Immunoblot assay and immunohistochemical analyses of primary and metastatic (synchronous and metachronous) CRAC (**i**–**m**). Synchronous metastatic (Mets) CRAC displayed lower expression and metachronous Mets displayed higher levels of HNF4α compared to primary CRAC. Scale bar = 100 μm.

HNF4α expression was evaluated in 148 CRAC samples, 11 TA samples, and 20 non-neoplastic epithelia tissue samples from CRAC patients. HNF4α immunostaining was detected in colonic epithelial cells, which exhibited nuclear expression. All non-neoplastic epithelia were negatively or weakly stained for HNF4α (Figure 3a). TAs showed faint nuclear expression, while HNF4α expression was upregulated in CRAC compared to TA (Figure 3b). Notably, metastatic CRAC showed a lower expression of HNF4α compared with primary CRAC (Figure 3c). HNF4α expression was elevated in moderately differentiated CRAC (Figure 3d) compared to poorly differentiated (Figure 3e) or poorly cohesive (Figure 3f) groups. Moreover, primary CRAC samples displayed significantly higher levels of HNF4α than TAs (*p* = 0.002). Interestingly, metastatic CRAC displayed markedly reduced levels of HNF4α relative to primary CRAC (*p* = 0.012) (Figure 3g). The expression of HNF4α was significantly higher in well- and moderately differentiated CRAC compared to poorly differentiated (including poorly cohesive) CRAC (Figure 3h).

Western blot for HNF4α was performed using five pairs of fresh frozen tissue samples of primary and metastatic CRAC stored at −80 °C in liquid nitrogen. The data revealed the differential expression of HNF4α between primary and metastatic CRAC tissue samples. Synchronous metastatic (Mets) CRAC displayed a lower expression compared to primary CRAC (*p* = 0.010); conversely, metachronous Mets CRAC displayed higher levels of HNF4α in relation to primary CRAC (*p* = 0.014) (Figure 3i). Among the 148 CRAC cases examined, three experienced synchronous and metachronous metastases following surgical resection. The expression of HNF4α was the lowest in synchronous metastasis compared to both primary and metachronous CRAC tissue samples (Figure 3j–m) (Supplementary Data S1).

### 3.4. Immunohistochemical Expression of HNF4α in Relation to Clinicopathologic Features and Survival of CRAC Patients

A total of 148 CRAC samples with clinicopathologic variables were investigated (Table 1). Patient ages ranged from 29 to 88 years, with a mean age of 63.3 years. HNF4α expression was negatively correlated with pathologic stage (I–II vs. III–IV), poor differentiation (including mucinous and poorly cohesive types), and nodal metastasis (*p* = 0.037, *p* = 0.008, and *p* = 0.003, respectively; Table 1).

**Figure 4:**
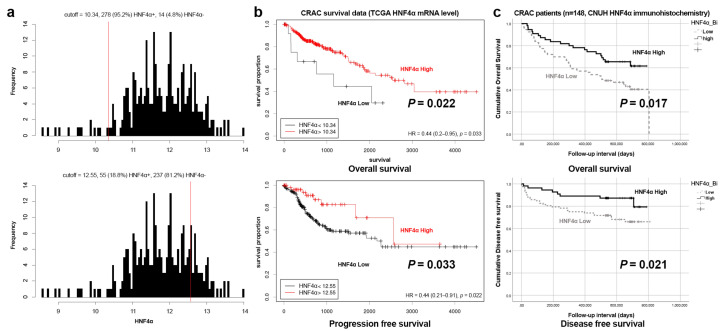
Kaplan–Meier survival curves of HNF4α expression in CRAC. (**a**) Determination of cutoff values (red lines) using the cutoff finder. (**b**) Overall and progression-free survival curves obtained with data from HNF4α mRNA level from the TCGA (*p* = 0.022 and *p* = 0.033). (**c**) Overall and disease-free survival curves obtained using HNF4α protein level data from CNUH (*p* = 0.017 and *p* = 0.021).

Overall and disease-free survival analyses were performed based on data from 329 patients in TCGA using mRNA levels of HNF4α and 148 patients in CNUH databases using protein levels of HNF4α (Supplementary Table S1 and Supplementary Data S2). The cutoffs were calculated using the Cutoff Finder tool (https://molpathoheidelberg.shinyapps.io/CutoffFinder_v1/ (accessed on 27 December 2024)), which established thresholds as 10.354 and 12.55 for the categorization of HNF4α expression into low and high levels (Figure 4a). The Kaplan–Meier survival curves revealed a significant correlation of HNF4α-low expression with reduced overall and progression-free survival rates (*p* = 0.022 and *p* = 0.033, respectively) in the TCGA group (Figure 4b) and decreased overall and disease-free survival (*p* = 0.017 and *p* = 0.021, respectively) in the CNUH cohort (Figure 4c).

Multivariate analyses were conducted using Cox’s proportional hazard model with all clinicopathologic variables with HNF4α expression. The results showed that a low expression of HNF4α serves as a significant prognostic factor associated with poor disease-free survival (*p* = 0.036) (Table 2). Multivariate analysis of overall survival using the Cox’s proportional hazard model did not yield statistically significant results in relation to HNF4α expression (*p* = 0.113).

### 3.5. HNF4α Expression in Relation to NF-κB, YAP, and EMT Markers in CRAC

**Figure 5:**
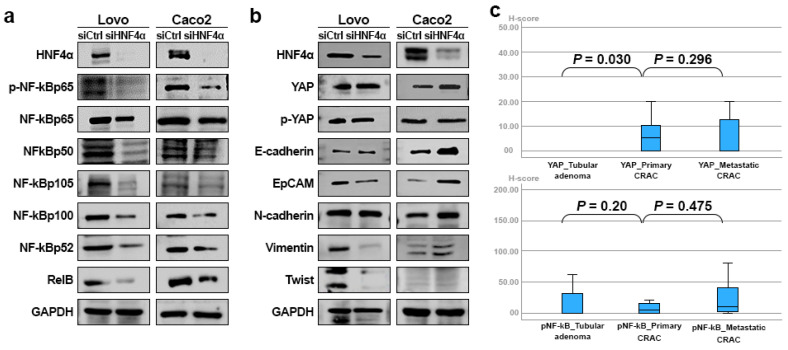
Immunoblot assay of the NF-κB, YAP, and EMT markers in CRAC cell lines (**a**) Decrease in NF-κB markers under conditions of HNF4α inhibition in LoVo and Caco2 cells. (**b**) Increase in YAP, E-cadherin, and N-cadherin under conditions of HNF4α inhibition in LoVo and Caco2 cells. Decrease in EpCAM and Vimentin and, conversely, increase in EpCAM and Vimentin in LoVo cells depleted of HNF4α. (**c**) Immunohistochemical changes in pNF-κb and YAP in tubular adenoma, primary CRAC, and metastatic CRAC.

To evaluate the effect of inhibition of HNF4α on signaling pathway proteins, Western blot analysis was employed. To this end, the canonical (p-p65, p65, p50, p105) and noncanonical (p100, p105, p52, RelB) NF-κb proteins, YAP and p-YAP, and EMT-related proteins (E-cadherin, EpCAM, N-cadherin, Vimentin, Twist) were examined following the regulation of HNF4α (Supplementary Data S3). The inhibition of HNF4α in LoVo and Caco2 cells induced a decreased tendency in NF-κB proteins related to the canonical and noncanonical pathways (*p* = 0.109, *p* = 0.285, *p* = 0.109, *p* = 0.109, *p* = 0.109, *p* = 0.109, respectively) (Figure 5a), alongside an increased tendency in YAP and the epithelial marker E-cadherin (*p* = 0.285 and *p* = 0.109) (Figure 5b). The inhibition of HNF4α in Lovo and Caco2 cells led to a decreased tendency in Twist protein levels (*p* = 1.000); however, the responses of EpCAM and Vimentin varied, whereby the levels of these proteins were decreased in LoVo and increased in Caco2 cells, without statistical significance.

Immunohistochemical analysis of YAP and p-NF-κB proteins was conducted on a cohort of 148 patients diagnosed with CRAC. Primary CRAC displayed significantly elevated levels of YAP than tubular adenomas (*p* = 0.030). However, the expression of HNF4α was not significantly different between metastatic and primary CRAC (*p* = 0.296) (Figure 5c). Additionally, p-NF-κB expression was not significantly different between adenoma and primary and metastatic CRAC samples (*p* = 0.2 and 0.475, respectively).

## 4. Discussion

HNF4α is a highly conserved member of the nuclear receptor superfamily of ligand-dependent transcription factors. HNF4α expresses in several epithelial cells that play a crucial regulatory role in cellular morphogenesis and function. The elevated expression of RNA and protein coupled with the rare frequency of mutations of HNF4α (0.65–1.68%, primarily amplification) in CRAC implicates the upregulation of HNFA as the key step in the carcinogenic progression of CRAC [12]. In this study, we evaluated HNF4α expression in CRAC using a panel of commercial cell lines and human tissue samples from patients. A subset of colon cancer cell lines displayed increased HNF4α expression, and immunohistochemical analysis revealed the upregulation of HNF4A expression during the process of carcinogenesis relative to normal and precancerous lesions. Accordingly, we propose that HNF4α plays a critical role in the early stages of colon carcinogenesis.

Previous studies highlighted the distinct roles of HNF4α isoforms generated by alternative (P1 and P2) promoters [22,24]. The findings suggested that the P1-isoform is suppressed by β-catenin in the early stages of colon tumorigenesis, whereas the P2-isoform supports oncogenic signalization by promoting CRAC cell survival and progression [24]. P1-HNF4α may function as a tumor suppressor, while P2-HNF4α plays an oncogenic role. In our study, a subset of CRAC cell lines and tissue samples from patients generally showed faint or weak P1, while strong P2-HNF4α protein expression was observed in the Western blot assay. During carcinogenesis, the upregulation of HNF4α in CRAC tissue samples was mainly in the form of P2-HNF4α protein and involved in the early phase of tumorigenesis. The evaluation of the effects of HNF4α modulation in colonic cancer cell lines revealed that the overexpression and inhibition of HNF4α were positively correlated with increased and suppressed cell migration, invasion, and wound closure, respectively, indicating that HNF4α plays an oncogenic role in CRAC cell invasiveness and migration.

Interestingly, the expression pattern of HNF4α did not correlate with CRAC aggressiveness and progression to metastasis. Metastatic CRAC displayed a significantly diminished expression of HNF4α compared with primary CRAC. Additionally, HNF4α expression was markedly lower in poorly differentiated relative to well- and moderately differentiated CRAC. The progression to dedifferentiation and metastasis of CRAC was related to the downregulation of HNF4α expression. This decrease was reversed in cases of metachronous metastasis (occurring six months after surgical resection). Patients with low-HNF4α expression in primary CRAC had poorer overall and disease- (or progression-) free survival outcomes, as supported by data from CNUH and TCGA. Conversely, elevated HNF4α expression was associated with a more favorable prognostic outlook.

The clinical significance of HNF4α expression appears ambivalent, since HNF4α is overexpressed in early carcinogenesis, and its expression is decreased during progression to metastatic CRAC. Similar results showed that HNF4α levels are lower in metastatic HCC and correlated with more aggressive clinical characteristics [20]. Furthermore, the loss of P1-HNF4α expression was documented in metachronous liver metastases associated with CRAC [30]. The variable expression of HNF4α over different stages of cancer progression highlights the significant challenges in developing targeted therapies for CRAC and suggests that HNF4α-targeting strategies may only benefit a specific subset of CRAC patients [31].

**Figure 6:**
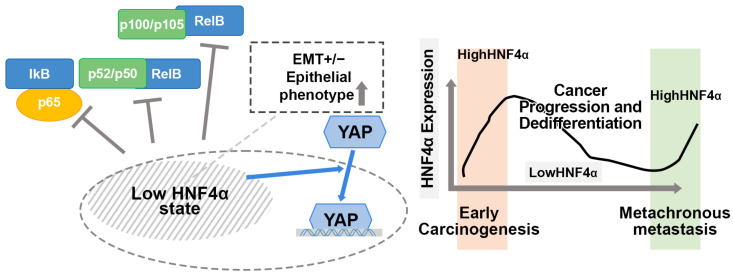
Hypothesis of HNF4α in relation to signaling pathways: low HNF4α state related to the suppression of NF-κb pathways, an increase in epithelial markers, and an increase in YAP levels. Changes in HNF4α expression in CRAC patients: (i) increased HNF4α expression during early carcinogenesis, (ii) lower expression during progression and dedifferentiation, and (iii) higher expression in metachronous metastasis.

HNF4α was identified as a key factor associated with EMT [29] and stemness linked to the Wnt/β-catenin pathway, metabolic regulation via NOTCH signaling, YAP/Hippo pathway, cell cycle modulation, and reactive oxygen species system in cancers [4,12,32]. HNF4α exerts an inhibitory effect on EMT through its effect on the Wnt/β-catenin signaling pathway via the upregulation of E-cadherin, along with the downregulation of β-catenin and Vimentin, which collectively contribute to **the** suppression of tumor growth and metastasis in HCC [19,33]. The current investigation focused on the effects of the regulation of HNF4α and associated changes in proteins related to NF-κb, YAP, and EMT. The suppression of HNF4α led to a decline in NF-κb protein levels from both canonical and noncanonical pathways. Additionally, a decrease in HNF4α was correlated to an increase in epithelial markers, while the expression of mesenchymal markers differed depending on the cell lines. Interestingly, the inhibition of HNF4α led to an increase in YAP levels. The expression of YAP was increased during carcinogenesis and cancer progression. Accordingly, we hypothesized that a low-HNF4α state of CRAC could contribute to the suppression of inflammatory NF-κb signaling and activation of the YAP/Hippo pathway for cancer progression and dedifferentiation (Figure 6). One plausible explanation for the ambivalent expression of HNF4α in CRAC is the differential interplay of related signaling pathways. Earlier studies interpreted the loss of HNF4α as an underlying mechanism for the regulation of cell identity under pathologic conditions associated with organ dysfunction [34]. Because of the lack of in vivo animal experiments and statistically insufficient data in relation to the signaling pathways, our study has limitation for revealing the effect of HNF4α, and further evaluation is required. However, our findings might provide insights that could contribute to a better understanding of the role of HNF4α in CRAC.

## 5. Conclusions

HNF4α was identified as a novel biomarker and a promising target for cancer therapy. The expression patterns and functions of HNF4α vary significantly across different types of cancers. Extensive insights into the expression and regulatory mechanisms of HNF4α in cancer progression and metastasis should enhance our understanding of its clinical significance. This study focused on the potential role of HNF4α in the progression of CRAC and the underlying molecular mechanisms. HNF4α plays a dual role in CRAC in that its overexpression is linked to early carcinogenesis and progression and loss of expression associated with the metastatic stage of the disease. Our findings collectively indicate that HNF4α expression varies according to the pathologic state of CRAC and activation of specific signaling pathways.

## Figures and Tables

**Table 1:** Correlation between immunohistochemical expression of HNF4α and clinicopathologic factors in patients with CRAC (*n* = 148).

Characteristics	Patients		HNF4α	
	No. (%)	Low	High	*p*
Sex				0.118
Male	93	54	39	
Female	55	39	16	
Age (mean)	63.3	63	63.8	0.614
Tumor size (mean, cm)	3.6	3.6	3.4	0.404
Differentiation				0.037
WD + MD	141 (95.3%)	86 (92.5%)	55 (100%)	
PD	7 (4.7%)	7 (7.5%)	0 (0%)	
Pathologic stage				0.008
I–II	87 (58.8)	47 (50.5)	40 (72.7)	
III–IV	61 (41.2)	46 (49.5)	15 (27.3)	
Nodal metastasis				0.003
Absent	90 (60.8)	48 (51.6)	42 (76.4)	
Present	58 (39.2)	45 (48.4)	13 (23.6)	
Distant metastasis				0.363
Absent	136 (91.9)	84 (90.3)	52 (94.5)	
Present	12 (8.1)	9 (9.7)	3 (5.5)	
Radiotherapy				0.554
Not done	137 (92.6)	87 (93.5)	50 (90.9)	
Done	11 (7.4)	6 (6.5)	5 (9.1)	
Chemotherapy				0.633
Not done	19 (12.8)	11 (11.8)	8 (14.5)	
Done	128 (87.2)	82 (88.2)	47 (85.5)	

WD, well differentiated; MD, moderately differentiated; PD, poorly differentiated.

**Table 2:** Outcomes of multivariate analysis of overall and disease-free survival in patients with CRAC (*n* = 148).

	Disease-Free Survival			Overall Survival		
	*p*	HR	95% CI	*p*	HR	95% CI
HNF4α (low vs. high)	0.036	0.407	0.176–0.943	0.113	0.642	0.371–1.111
Sex (male vs. female)	0.233	1.547	0.755–3.169	0.216	0.721	0.430–1.211
Age (under 66 vs. over 66)	0.398	0.743	0.373–1.479	0.000	2.907	1.701–4.968
Size (<4cm vs. ≥4cm)	0.830	1.087	0.507–2.331	0.775	0.920	0.519–1.629
Differentiation (WD + MD vs. PD)	0.112	2.609	0.801–8.501	0.964	0.975	0.332–2.867
Stage (I + II vs. III + IV)	0.139	9.103	0.489–169.453	0.674	0.694	0.127–3.804
Nodal metastasis (Absent vs. Present)	0.212	0.163	0.009–2.823	0.185	3.039	0.587–15.743
Distant metastasis (Absent vs. Present)	0.295	0.337	0.044–2.581	0.007	3.271	1.393–7.682
Radiotherapy (Not done vs. Done)	0.000	7.789	0.324–18.725	0.009	2.860	1.294–6.325
Chemotherapy (Not done vs. Done)	0.637	0.789	0.261–2.379	0.651	0.837	0.387–1.810

## Data Availability

The data presented in this study are available on request from the corresponding author.

## Supplementary Materials

The following supporting information can be downloaded at: https://www.mdpi.com/article/10.3390/cells14080599/s1, Figure S1: Our study flow chart; Table S1: Correlation between HNF4α mRNA level and clinical factors of CRAC from the TCGA data (n = 292); Supplementary data S1: WB Figure 3 Supplementary data, Supplementary data S2: CRAC TCGA Supplementary data and Supplementary data S3: Figure 5 WB Supplementary data.
