# Supplementary material for: Dual Role of HNF4α in Colorectal Adenocarcinoma During Carcinogenesis and Metastasis
Source: Cells. 2025 Apr 15;14(8):599. doi: 10.3390/cells14080599 (PMC12025726; doi:10.3390/cells14080599)
Supplement: Supplementary file 1 [file cells-14-00599-s001.zip › cells-3500566-supplementary.pdf]

Supplementary Materials

Dual Role of HNF4α in Colorectal Adenocarcinoma During Carcinogenesis and Metastasis

Ju Seok Kim [1,†], Kyung-Hee Kim [2,†], Jun Young Heo [3,4,5], Min Kyung Choi [2] and Min-Kyung Yeo [2,4,5,*]

1 Department of Internal Medicine, Chungnam National University School of Medicine, Daejeon 34134, Republic of Korea; showsik@cnuh.co.kr
2 Department of Pathology, Translational Immunology Institute, Chungnam National University School of Medicine, Daejeon 34134, Republic of Korea; phone330@cnu.ac.kr (K.-H.K.); mk6214@cnu.ac.kr (M.K.C.)
3 Department of Biochemistry, Chungnam National University School of Medicine, Daejeon 34134, Republic of Korea; jyheo@cnu.ac.kr
4 System Network Inflammation Control Research Center, Chungnam National University, Daejeon 34134, Republic of Korea
5 Department of Medical Science, Chungnam National University School of Medicine, Daejeon 34134, Republic of Korea
* Correspondence: mkyeo83@cnuh.co.kr or mkyeo83@gmail.com; Tel.: +82-42-580-8238; Fax: +82-42-581-5233
† These authors contributed equally to this work.

Supplementary Table S1. Correlation between HNF4α mRNA level and clinical factors of CRAC from the TCGA data (n=292).

Characteristics	Patients	HNF4α		
	No. (%)	Low	High	P
Sex				0.084
Male	161 (55.3)	116 (52.5)	45 (64.3)	
Female	130 (44.7)	105 (47.5)	25 (35.7)	
Age (mean)	292 (65.0)	221 (64.6)	70 (65.9)	0.278
Pathologic stage				0.352
I	46 (16.4)	35 (16.4)	11 (16.7)	
II	110 (39.3)	90 (42.1)	20 (30.3)	
III	82 (29.3)	59 (27.6)	23 (29.3)	
IV	42 (15.0)	30 (14.0)	12 (15.0)	
Death during follow-up period				0.099
Absent	215 (73.9)	158 (71.5)	57 (81.4)	
Present	76 (26.1)	63 (28.5)	13 (18.6)	
Recurrence during follow-up period				0.035
Absent	237 (81.4)	174 (78.7)	63 (90.0)	
Present	54 (18.6)	47 (21.3)	7 (10.0)	
Recurrence during follow-up period				0.060
Absent	207 (71.1)	151 (68.3)	56 (80.0)	
Present	84 (28.9)	70 (31.7)	14 (20.0)	

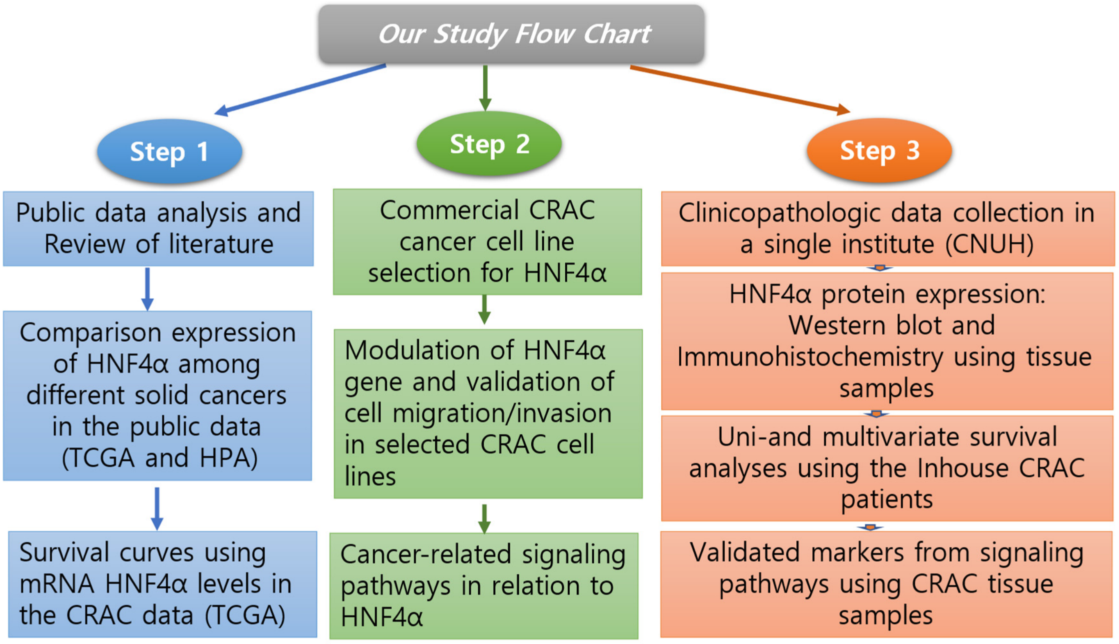

Supplementary Figure S1. Our study flow chart.
